# A high-throughput colorimetric assay for detection of *Schistosoma mansoni* viability based on the tetrazolium salt XTT

**DOI:** 10.1186/s13071-017-2240-3

**Published:** 2017-06-21

**Authors:** Pedro Henrique Nascimento Aguiar, Núbia Monteiro Gonçalves Soares Fernandes, Carlos Leomar Zani, Marina Moraes Mourão

**Affiliations:** 1 Laboratório de Helmintologia e Malacologia Médica, René Rachou Research Center, FIOCRUZ, Belo Horizonte, Minas Gerais Brazil; 2 Laboratório de Química dos Produtos Naturais, René Rachou Research Center, FIOCRUZ, Belo Horizonte, Minas Gerais Brazil

**Keywords:** *Schistosoma mansoni*, XTT, Drug screening, High - throughput screening

## Abstract

**Background:**

*Schistosoma mansoni* is a trematode parasite that causes schistosomiasis, one of the most prevalent neglected tropical diseases, leading to the loss of 2.6 million disability-adjusted life years. Praziquantel is the only drug available, and new drugs are required. The most common strategy in schistosomiasis drug discovery is the use of the schistosomula larval-stage for a pre-screen in drug sensitivity assays. However, assessing schistosomula viability by microscopy has always been a limitation to the throughput of such assays. Hence, the development of validated, robust high-throughput in vitro assays for *Schistosoma* with simple readouts is needed. Here, we present a simple and affordable alternative to assess schistosomula viability. The method employed is based on the hydrosoluble tetrazolium salt XTT which has been widely used in other organisms but has never been used to drug screen in schistosomes.

**Results:**

We showed that schistosomula reduce XTT salt to a coloured formazan product and that absorbance levels reflected the viability and parasites number. This XTT viability assay was validated for high throughput screening of compounds in schistosomula, and dose-response curves of compounds could be reproduced.

**Conclusions:**

We conclude that the XTT viability assay could be applied for the screening of large compounds collections in *S. mansoni* and accelerate the identification of novel antischistosomal compounds.

## Background

The helminth parasite *Schistosoma mansoni* is the causative agent of schistosomiasis, a parasitic disease that afflicts over 258 million people in tropical and subtropical areas [1]. Currently, there is no vaccine to prevent schistosomiasis transmission, and treatment relies exclusively on one drug, praziquantel (PZQ). Despite its high efficacy and low cost, the mass administration and reliance on a single drug increases the risk of the development of drug resistance. In fact, cases of significantly reduced susceptibility to PZQ have already been reported in field and laboratory isolates [2–4]. Hence, drug discovery in schistosomiasis is still of great relevance, and robust high throughput parasite screening techniques are urgently needed.

Microscopic assessment of morphology and motility has always been considered the gold standard for schistosomula viability evaluation [5, 6]. However, this method can be subjective, causing conflicting results among different laboratories as it relies on a trained observer to interpret parasite phenotype. Moreover, it is a very laborious and time-consuming technique that limits the throughput of drug screening projects and consequently delays the identification of lead compounds. Such limitations have compelled the scientific community to search for new high-throughput screening (HTS) assays for *Schistosoma,* and indeed, many techniques have recently been developed [7]. Some of these methods measure parasite motility, such as the WormAssay [8], while others make use of the automated image-based classification for parasite phenotype characterization [9, 10].

Dye-based methods are the most common choice for viability assays, as they can be read by an automatic plate reader in a fast, simple and practical manner. Moreover, such assays require little training and do not need highly qualified professionals to execute. Some fluorescent and luminescent assays have been attempted for assessing schistosomula viability. Mansour & Bickle [11] proposed the use of Alamar Blue to determine schistosomula viability. However, this fluorescent indicator of metabolic activity showed inconsistency for compounds that were not lethal but only reduced parasite viability. Another fluorescent assay developed was the dual-marker bioassay using propidium iodide (PI) and fluorescein diacetate (FDA), a duplex assay that allows the simultaneous assessment of schistosomula viability and cytotoxicity [12]. This method has shown promising results as the use of fluorophores that stain independently dead or live schistosomula allowed the quantification of viable parasites across a range of viability endpoints. Nevertheless, the number of parasites per well required to achieve a good correlation between the signal and viable schistosomula concentration is too high and presents a limit to the throughput of the assay [7]. A fluorometric kit that measured lactate levels was also tested for drug screening in *S. mansoni* by Howe et al. [13]. This byproduct of glycolysis is secreted via aquaglyceroporins from schistosomula and adult worms and could be used as an indicator of metabolic activity. Their research showed that this assay successfully measured parasite number and viability through quantification of lactate levels in parasite culture supernatant. However, the requirement of removing the supernatant from parasite culture plates (without aspirating schistosomula) and the processing steps afterwards reduces the throughput of the assay. Recently, Panic et al. [7] performed a testing and comprehensive revision of marker-based assays on *S. mansoni* to identify a suitable method for HTS of schistosomula. Resazurin (Alamar Blue), Vybrant® and CellTiter-Glo® provided the best signal correlation with schistosomula viability. However, only the CellTiter-Glo® Luminescent Kit was capable of accurately determining IC_50_ values of some antischistosomal drugs. This result is in accordance with Lalli et al. [14], who first proposed the use of the CellTiter-Glo® Kit to determine schistosomula viability.

Fluorescent/luminescent assays present some disadvantages when compared to colorimetric assays. First, they may require the use of black or white opaque walled culture plates to avoid high background or crosstalk between adjacent wells, and in some cases, plates with optically clear bottoms are also necessary. Such culture plates present much higher cost when compared to the standard clear plates that are used in colorimetric methods. Secondly, fluorophores and luminescent markers are more expensive than colorimetric dyes. Additionally, some fluorescent/luminescent assays may require high-sensitivity microplate readers that present advanced well-scanning features to accurately measure signals in non-homogeneous samples, such as the PI and FDA whole organism-based assay [12], where the marker stains the parasite itself which is unevenly distributed across the well. Panic et al. [7] and our group have failed to reproduce the PI and FDA assay using a simple microplate reader. Thus, the lack of reproducibility might be due to the type of plate reader, and the use of this methodology might be restricted to groups with access to this high-cost equipment. These disadvantages prompted us to investigate an affordable, easy-to-use, and fast colorimetric assay to measure schistosomula viability in a high throughput assay that could be employed in drug screening platforms.

Colorimetric assays based on the reduction of tetrazolium salts have been widely used for quantification of cell viability and proliferation [15]. The colorimetric method is based on the reduction of 3-(4,5-dimethylthiazol-2-yl)-2,5-diphenyltetrazolium bromide (MTT) to a highly coloured formazan product and was first developed by Mosmann [16]. The reduction of MTT produces an insoluble formazan product, which requires the aspiration of culture media and the addition of organic solvents to dissolve the crystals for absorbance measurements. These processing steps are time-consuming and may be difficult to execute when working with non-adherent cells or organisms. Roehm [17] proposed the use of the hydrosoluble formazan XTT (sodium-2,3- bis-[2-methoxy-4-nitro-5-sulfophenyl]-2H–tetrazolium-5-carboxanilide) to overcome this MTT assay issue. The bioreduction of XTT yields a coloured formazan product that is water soluble, and furthermore, the use of electron coupling agents, such as phenazine methosulfate (PMS), can accelerate the reduction of the tetrazolium salt allowing for better results with shorter incubation periods. In addition to mammalian cells, the XTT assay has shown applicability to bacteria [18], fungi [19], and *Leishmania* [20] assays, but to date, no research group has proposed the use of XTT to measure *Schistosoma* viability. In the present work, we developed a schistosomula viability assay based on XTT and validated this high - throughput screening assay using compounds with previously described antischistosomal activity. This is the first report of the application of the hydrosoluble tetrazolium salt XTT to assess *Schistosoma* viability.

## Methods

### Parasites

*Schistosoma mansoni* cercariae (LE strain) were harvested from the intermediate host *Biomphalaria glabrata* at René Rachou Research Center, FIOCRUZ. Newly transformed schistosomula (NTS) were obtained by mechanical in vitro transformation of cercariae using a protocol adapted from a previously described method [21]. Briefly, the cercarial suspension was distributed in 50 ml conical tubes and cooled on ice for 60 min. Cercariae were concentrated by centrifugation at 1000× *g* for 3 min at 4 °C, followed by resuspension in Medium 199 (without phenol red; Sigma-Aldrich, St. Louis, USA) supplemented with 100 U/ml penicillin and 100 μg/ml streptomycin. Tails were detached by passing cercariae 4 times through a 22 G needle. Parasites were incubated at 37 °C and 5% CO_2_ for 60 min before proceeding with washing steps. Schistosomula were separated from cercarial tails by 10 cycles of washing and sedimentation, and microscope examination was used to assess number and quality of purified parasites. NTS were cultured in Medium 199 (without phenol red) supplemented with 2% inactivated Fetal Bovine Serum (GIBCO, Waltham, USA), 100 U/ml penicillin and 100 μg/ml streptomycin (GIBCO) at 37 °C and 5% CO_2_ for 24 h prior to experiments. Parasites were plated into clear flat-bottom 96-well tissue culture plates in 200 μl media or 384-well plates in 50 μl media depending on the assay.

### XTT schistosomula viability assay

Tetrazolium salt XTT (sodium 3′-[1-[(phenylamino)-carbony]-3,4-tetrazolium]-bis(4-methoxy-6-nitro)benzene sulfonic acid hydrate) was purchased from Sigma-Aldrich. The XTT viability assay protocol was adapted from a previous version described for normal activated T cells [17]. XTT solution was prepared by dissolving 1 mg/ml in Medium 199 (without phenol red; Sigma-Aldrich) and warming to 55 °C in a water bath until completely dissolved. The electron coupling reagent phenazine methosulfate (PMS; Sigma-Aldrich) was used to enhance XTT reduction. PMS was dissolved at 0.383 mg/ml in phosphate buffered saline (PBS). Both XTT and PMS solutions were filtered through 0.2 μm pore size membrane aliquoted and stored at -20 °C until use. NTS were cultured for 48 h before each XTT viability assay. Thereafter, an XTT labelling mixture was prepared by mixing XTT and PMS solutions in a 50:1 ratio and 40 μl was added to each well of the 96-well plates, which contained 200 μl of NTS suspension, or 10 μl to each well of the 384-well plates, which contained 50 μl of NTS suspension. The standardisation of the method was carried out as follows: four different concentrations of parasites were used (100, 200, 300 and 400 parasites per well) and the absorbance readouts were performed at intervals of 4, 6 and 24 h of incubation with XTT at 37 °C and 5% CO_2_. The absorbance at 450 nm (reference wavelength of 690 nm) was determined using SpectraMax M5 microplate reader (Molecular Devices, CA, USA). Absorbance values obtained were used to determine schistosomula viability using the following equation:

$$ \%\mathrm{Viability}=\frac{\left(\mathrm{Sample}\hbox{-} \mathrm{Negative}\ \mathrm{control}\right)}{\left(\mathrm{Positive}\ \mathrm{control}\hbox{-} \mathrm{Negative}\ \mathrm{control}\right)}\times 100 $$

where “Sample” is the absorbance measured from each well containing parasites tested with compounds, “Negative control” represents the average absorbance measured from heat-killed parasites (incubated at 65 °C for 10 min) and “Positive control” is the average absorbance measured from untreated parasites. Parasite viability was checked throughout the entire process by microscopy assessment to investigate if the dye does not damage the NTS.

During most of our experiments, we performed counting of parasites plated during XTT assays, and parasite number was determined for some wells that presented absorbance levels higher than the average, below the average, and near the average values. Those numbers revealed that the XTT assay could tolerate approximately 25% variation without producing a difference in absorbance values.

### XTT validation experiments

Experiments following the HTS Assay Validation section from the NIH Assay Guidance Manual [22] were conducted to validate the XTT schistosomula viability assay. A plate uniformity study was performed to assess uniformity and separation of signals, and to ensure that the signal window is adequate to detect active compounds during a screen. Three types of signals were measured: the “High” signal, representing the maximum signal, obtained with untreated parasites in the presence of 0.1% dimethyl sulfoxide (DMSO) alone; “Min” signal, to measure the background signal, generated by heat-killed parasites; and the “Mid” signal, to estimate the signal variability between the maximum and minimum signals, obtained by reading the absorbance from wells containing a 1:1 mixture of untreated and heat-killed parasites. The three preparations of parasites were distributed in 96-well (200 NTS in 200 μl/well) or 384-well (100 NTS in 50 μl/well) culture plates following the Interleaved-signal format plate layout, which consisted of a combination of “Max”, “Mid” and “Min” signals on all plates varying systematically, so that on every experiment all signals are measured on every position of all plates. Parasites were incubated for 48 h at 37 °C and 5% CO_2_, and then the XTT viability assay was performed as described above. Absorbance values were added in the Data Analysis Excel (Microsoft Corporation, CA, USA) Templates and instructions available at https://www.ncbi.nlm.nih.gov/books/NBK83783/. The Data Analysis Templates performed the statistical tests required to check if the assay meets the criteria: (i) mean; (ii) standard deviations (the SD for the “Min” signal should be smaller than the SD for the “Max” and “Mid” signals); (iii) coefficient of variation (all “Max” and “Mid” signals should have CV’s less than 20%), (iv) signal window (SW ≥ 2), and (5) Z’ factor calculation (Z’ ≥ 0.4). The presence of drift or edge effects were also checked in the data analysis Excel templates.

### Drug sensitivity assay

To assess if the XTT viability assay could be used to determine IC_50_ values in a dose-response curve, six compounds with previously reported activities were tested. Compounds were resuspended in DMSO to 10 mM stock concentration, and then serially diluted in Medium 199 (supplemented as stated above) as follows: 40, 20, 10, 5, 2.5, 1.25 and 0.625 μM for mefloquine [13] and Ivermectin [23]; 80, 40, 20, 10, 5, 2.5, 1.25 and 0.625 μM for JQ1 and curcumin [24]; 10, 5, 2.5, 1.25, 0.625, 0.312, 0.156 and 0.078 μM for amphotericin; and 1000, 500, 250, 125, 62.5, 31.25, 15.62 and 7.81 μM for praziquantel [13]. Parasites were incubated with compounds for 48 h after which the XTT viability assay was performed as described above. Schistosomula viability after treatment with drugs was determined by the equation described previously. Positive control used here was the average absorbance measured from parasites treated with vehicle alone (0.1% DMSO), and the negative control was the average absorbance measured from heat-killed parasites (as stated above).

To investigate if compound coloration could interfere with the colorimetric XTT assay, curcumin and amphotericin dose-response curves were also performed in an alternative protocol with a washing step prior to XTT viability assay, which consisted of removing 50% of culture supernatant containing the compounds and replacing with new medium without compound followed by a quick spin (600× *g*). This washing step was repeated three times.

The XTT viability assay for each compound was accompanied by a microscopy assay to compare the two methods. Parasite viability assessment by microscopy was performed by staining with 5 μg/ml propidium iodide [25], and counting dead and live parasites using a Carl Zeiss Axio Observer fluorescence inverted microscope equipped with a Rhodamine (536 excitations) filter.

### Statistical analysis

The statistical analyses in this work were performed using the GraphPad Prism 5.0 program (GraphPad Software Inc., CA, USA), except the analyses performed in the Plate Uniformity Study that was performed in Microsoft Excel 2010 (Microsoft Corporation, CA, USA). Data are presented as the mean ± standard deviation (SD). Results were analysed for significant differences using ANOVA or Student’s *t*-test. Statistical tests used are described in each figure legend. The level of significance was set at *P* < 0.05.

## Results

### The formazan product absorbance correlates with the number of viable schistosomula

**Fig. 1:**
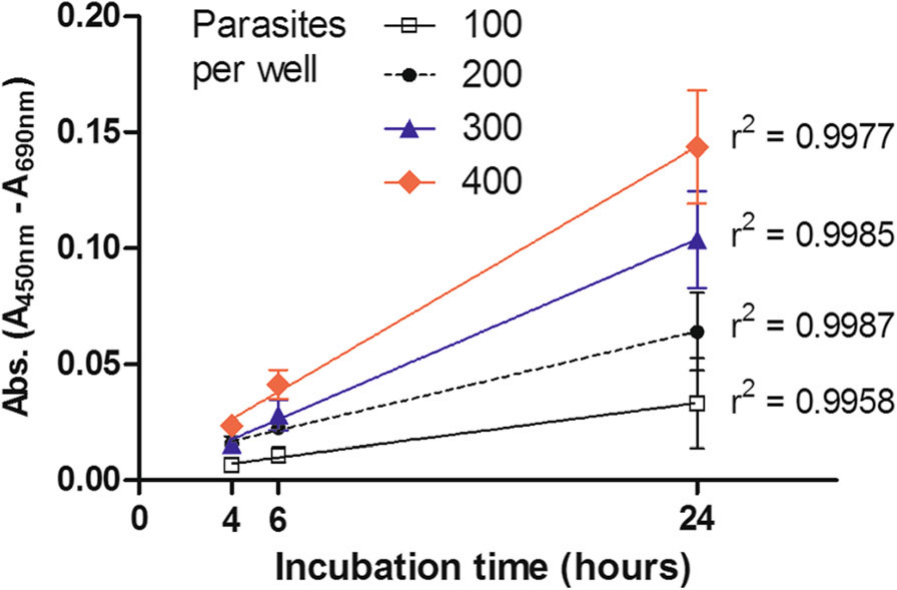
Absorbance levels of formazan product for different parasites concentration over time. Mechanically transformed schistosomula were distributed in 96-well plates at the concentrations of 100, 200, 300 and 400 parasites per well. Schistosomula were cultured for 48 h before addition of XTT labeling mixture. Parasites were incubated with XTT labelling mixture for 4, 6 and 24 h before absorbance measurements. Linear regression *r* ^2^ values are indicated on the graph

The first parameter assessed to optimise the XTT viability assay was the incubation period required to obtain a maximal reproducibility of parasite number and viability. For this, a standardisation step was performed, which included different concentrations of parasites and incubation periods. Figure 1 demonstrates that there is a good correlation between signal and incubation time for all four concentrations of parasites tested. The optimal incubation period to distinguish between parasite concentrations was 24 h as demonstrated (Table 1).

**Table 1:** Results for statistical analysis of XTT viability assay with different incubation periods using Two-way ANOVA with Bonferroni *post-hoc* test between each replicate (*n* = 6)

Incubation time (h)	Replicates compared					
	100 *vs* 200	100 *vs* 300	100 *vs* 400	200 *vs* 300	200 *vs* 400	300 *vs* 400
4	ns	ns	ns	ns	ns	ns
6	ns	ns	*t*_(5)_ = 3.024, *P* = 0.0293	ns	ns	ns
24	*t*_(5)_ = 3.071, *P* = 0.0278	*t*_(5)_ = 7.015, *P* = 0.0009	*t*_(5)_ = 10.98, *P* = 0.0001	*t*_(5)_ = 3.944, *P* = 0.0109	*t*_(5)_ = 7.908, *P* = 0.0005	*t*_(5)_ = 3.964, *P* = 0.0107

*Abbreviation*: *ns* not significant

**Fig. 2:**
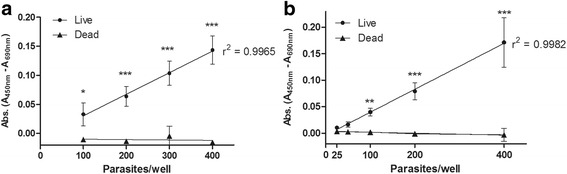
Correlation of absorbance levels to parasites concentration in 96-well (**a**) and 384-well (**b**) plates formats after 24 h incubation with XTT labeling mixture. Linear regression *r* ^*2*^ values are indicated on the graph and statistical analysis using Two-way ANOVA with Bonferroni *post-hoc* test between each replicate (*n* = 6). Significance values are represented by asterisks: **P < 0.01; ***P < 0.001

After determining the best incubation time for our experiments, we tested the assay sensitivity in two plate formats: 96-well plate for medium throughput and 384-well plate for high throughput format. First, both plates were assayed with increasing number of parasites per well. Linear regression analysis showed that there is a strong linear relationship between formazan absorbance and live parasite number (Fig. 2). In the 96-well plate format, a high correlation (*r* ^2^ = 0.9965, *P* = 0.0017) was found when analysing absorbance of wells containing from 100 to 400 live schistosomula (Fig. 2a). A very strong correlation between formazan absorbance and parasite number was also observed in the 384-well plate format (*r* ^2^ = 0.9982, *P* < 0.0001) in which we measured the absorbance of wells containing from 25 to 400 live schistosomula (Fig. 2b). The absorbance measured for heat killed parasites was approximately zero in all conditions. Formazan absorbance levels for live parasites only achieved levels high enough to differentiate them from dead parasites on wells containing more than 200 schistosomula (*t*_(5)_ = 6.003, *P* = 0.0018) in the 96-well plate format, and 100 schistosomula (*t*_(5)_ = 3.9940, *P* = 0.0104) in the 384-well plate format. For this reason, we have set our assays with 200 parasites per well in the 96-well plate format and 100 parasites per well in the 384-well plate format for subsequent experiments.

**Fig. 3:**
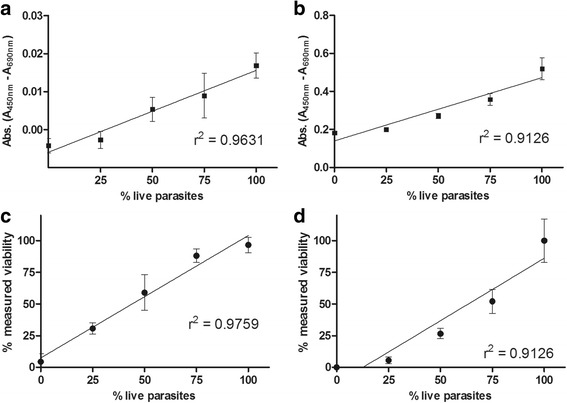
Correlation of absorbance levels to viable parasites concentration in 96-well (**a**) and 384-well (**b**) plates formats. Schistosomula viability was calculated from absorbance measured on 96-wells plates (**c**) and on 384-wells plates (**d**). Linear regression *r* ^2^ values are indicated on the graph

Next, we evaluated the assay sensitivity to detect possible drug hits that reduce parasite viability but do not kill 100% of the schistosomula. To investigate this, we prepared samples with varying percentages of live (untreated) and dead (heat-killed) schistosomula by mixing different volumes of both preparations in 96- and 384-well plates. Formazan absorbance values increased with the percentage of live parasites in both plate formats (Fig. 3a, b), and this allowed for the determination of parasite viability using the equation described in the Methods section (Fig. 3c, d). Once again, there was a high correlation between the viability measured through formazan product absorbance and the percentage of live parasites in the 96-well (*r* ^2^ = 0.9759, *P* = 0.0016) and 384-well (*r* ^2^ = 0.9126, *P* = 0.0113) plate formats, which was also confirmed by PI visual readouts.

### Validation of XTT viability assay

**Fig. 4:**
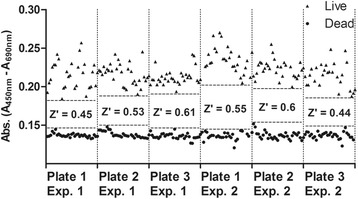
Plate uniformity study performed with different parasites preparations: DMSO treated parasites (Live); heat killed parasites (Dead). Parasites were distributed in three plates following the interleaved signal format in two independent experiments (templates available at https://www.ncbi.nlm.nih.gov/books/NBK83783/). The Z’ factor for each plate is represented in the graph

As the XTT assay was previously established in other organisms, we performed a 2-day Plate Uniformity study. This uniformity assay consisted of distributing three different parasite preparations eliciting different signal intensity (Max, Mid, and Min) in three plates for each day following layouts with a proper statistical design. The assay demonstrated good uniformity across the 6 plates, and no drift or edge effects were observed (Fig. 4). Analyses performed using the Excel Templates provided by the NIH Assay Guidance Manual have shown that all plates in both 96- and 384-well formats passed all the acceptance criteria, presenting Z’ factor score above 0.4 and Signal Window higher than 2 (Table 2), as recommended by previous authors [22, 26]. Raw signals were sufficiently tight with significant separation between Max and Min signals, which are adequate conditions to detect active compounds during a drug screen.

**Table 2:** Statistical analysis of data from the 12 plates of all plate uniformity experiments in both 96- and 384-well plates formats

Format	Plate	Z’ factor	Signal window
96-well	Plate 1/Experiment 1	0.45	2.75
	Plate 2/Experiment 1	0.53	4.38
	Plate 3/Experiment 1	0.61	6.25
	Plate 1/Experiment 2	0.55	4.75
	Plate 2/Experiment 2	0.6	6.35
	Plate 3/Experiment 2	0.44	3.07
384-well	Plate 1/Experiment 1	0.5	3.6
	Plate 2/Experiment 1	0.51	3.94
	Plate 3/Experiment 1	0.5	3.58
	Plate 1/Experiment 2	0.57	5.17
	Plate 2/Experiment 2	0.44	3.47
	Plate 3/Experiment 2	0.53	4.66

### The XTT viability assay allows IC_50_ determination of antischistosomal compounds

**Fig. 5:**
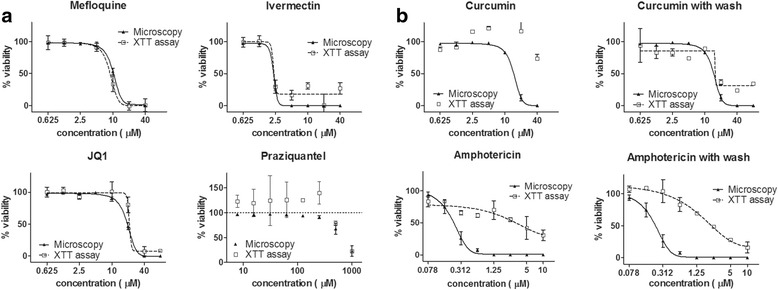
Dose-response curves of schistosomula exposed to compounds with reported activity against *S. mansoni* (**a**; mefloquine, ivermectin, JQ1, praziquantel) generated by the XTT viability assay and microscopy assessment. Microscopic counts and XTT viability assay started 48 h post-treatment. (**b**) Comparison of the readouts of the XTT viability assays of compounds that present yellow coloration (curcumin and amphotericin) performed without or with a washing step

After validation of the high throughput performance characteristics of the XTT assay, we performed drug sensitivity experiments to investigate the efficacy of the assay in determining schistosomula viability in the presence of known schistosomicidal compounds. These compounds were: the antimalarial drug mefloquine (MFQ) [27]; the antihelmintic Ivermectin (IVM) [28, 29]; the bromodomain inhibitor JQ1 [30]; and the reference drug praziquantel (PZQ). The positive and negative controls average absorbance values were used to calculate parasites viability. The XTT viability assay reproduced microscopy results for MFQ, IVM, JQ1 and PZQ (Fig. 5a). IC_50_ values for MFQ, IVM and JQ1 were successfully determined using both assays (Table 3), and PZQ only affected parasites viability in higher concentrations, as previously reported for this larval stage [13, 14].

**Table 3:** IC_50_ values (± SD) of compounds with reported activity against *S. mansoni* generated with XTT viability assay compared to values obtained by microscopy analysis

Compound	IC_50_ (μM)	
	XTT Assay	Microscopy
Mefloquine	8.9 ± 0.6	10.2 ± 0.2
Ivermectin	2.2 ± 0.1	2.3 ± 0.1
JQ1	21.2 ± 2.9	19.6 ± 0.3
Praziquantel	~ 588^a^	~ 472^a^
Curcumin	13.0 ± 5.7	14.7 ± 0.3
Amphotericin	2.4 ± 0.1	0.2 ± 0.04

^a^SD not determined

To evaluate if the coloration of compounds could interfere with the formazan product absorbance measurements, we included two coloured compounds with previously reported schistosomicidal activity, curcumin [24] and amphotericin [6]. Both compounds coloration produced high absorbance values which were translated into curves for the XTT viability assay that were not in accordance with microscopy analysis results (Fig. 5b). To overcome this issue, we performed drug sensitivity assays for curcumin and amphotericin including a washing step before the addition of XTT labelling mixture. The resulting dose response curve for curcumin reveals that the removal of the compound from the supernatant was efficient to stop the compound coloration interference in the XTT assay. However, the dose-response curve for amphotericin produced with the XTT assay after the washing steps was still different from the observed by microscopy assessment (Fig. 5b; Table 3).

## Discussion

In the present study, we present the schistosomiasis research community with the alternative of a “just-add”, easy-to-use and affordable colorimetric marker-based assay to determine schistosomula viability in drug screening. Schistosomiasis is one of the most prevalent neglected tropical disease causing more than 2.6 million disability-adjusted life years (DALYs) lost [31]. Drug discovery research in schistosomiasis is still of great importance as chemotherapy relies only on PZQ, and drug resistance poses a real threat [4]. Furthermore, the availability of the *Schistosoma mansoni* genome sequence [32] has provided insights into this parasite biology and opened new possibilities for potential drug target identification. Thus, new HTS methods are necessary to overcome the schistosome phenotypic screening bottleneck in the search for novel antischistosomal drugs.

Our work shows that the use of the colorimetric marker XTT applies to a *Schistosoma* viability assay. Here we showed that the formazan product absorbance values present a linear correlation with the number and viability of schistosomula (Figs. 2 and 3). We have established the optimal conditions for assessing parasites viability in a medium to high throughput assay (96- and 384-well plate formats). The method standardised in our laboratory was validated following the guidelines from the NIH HTS Assay Validation Manual [22] and passed all criteria required for a high throughput screening of compound libraries. Moreover, our results show that the XTT marker-based assay can reproduce the IC_50_ of antischistosomal compounds obtained by microscopy, considered the gold standard method (Fig. 5, Table 3).

The pursuit of a simple, precise and affordable marker-dye based assay for *S. mansoni* viability assessment in drug screening has been addressed by many researchers, with varying degrees of success. Peak and colleagues [12] developed a fluorescent duplex assay that employed PI and FDA to stain dead and live parasites respectively. While they achieved good results with high signal correlation to viable parasites, other research groups (including ourselves) could not reproduce their results [7]. The problem with this methodology is that it relies on markers that stain the schistosomula themselves, and the plate reader used must be equipped with a full well scanning feature to enable the reproducible read of non-homogenous samples such as the schistosomula culture, that may be unevenly distributed across the well-bottom. Therefore, the number of parasites required to achieve good signal correlation may be too high when using most common plate readers.

Spectrophotometric measurement involving markers that assess elements released into the medium may perform better than assays based on markers that stain parasites themselves [7]. Most recently, Lalli et al. [14] proposed the use of a luminescent viability kit (CellTiter-Glo®) to determine schistosomula viability through quantitation of ATP and showed this methodology can be used to assess schistosomula response in drug sensitivity assays. Their results were later reproduced by Panic et al. [7]. Nevertheless, a lack of sensitivity was observed while analysing compounds that damage but may not kill the worms, and drug dose-response results have shown a two-fold variation when compared to microscopic readouts [7].

The CellTiter-Glo® kit and the method proposed here using XTT rely on quantitation of metabolic activity to assess the number of viable parasites. Consequently, the number of parasites added to each well must be very consistent to ensure reproducible results. Indeed, while analysing our preliminary experiments we identified outliers in absorbance values that were accounted for by pipetting error after verification under the microscope (data not shown). The error was circumvented by the use of multichannel pipettes and thoroughly homogenising the parasites suspension before dispensing. Lalli et al. [14] employed a multi-drop dispenser to distribute a more uniform number of parasites within each assay, whereas Panic et al. [7] did not, which could explain the lack of sensitivity they reported for the CellTiter-Glo® kit.

Problems with compound interference in the detection method are common in absorbance and fluorescence assays, as some compounds may be fluorescent or coloured, leading to false negative or false positive results [33]. To investigate if this issue could interfere with the XTT viability assay we introduced in our drug sensitivity tests two compounds that present similar coloration to the formazan product and could absorb in the same wavelength, curcumin and amphotericin. The results obtained with both compounds, using absorbance values and microscopic evaluations, were discrepant (Fig. 5). This result shows that, indeed, coloration of the compounds can interfere with the test results. Counterassays can be designed to identify or minimise the interference from the compound library. In our case, we performed a washing step replacing the medium containing coloured compound before the addition of the XTT labelling mixture. The measure was effective for curcumin but not for amphotericin, showing that this could be a limitation for the assay. However, during a recent drug screening project in which our research group participated, among the 363 compounds tested, coming from diverse sources, only 3.9% presented a coloured yellowish tone and were active against schistosomula in a primary screening (Aguiar, unpublished results). This indicates that only a small percentage of compounds could produce ambiguous results and would need confirmation under the microscope. Nevertheless, the assay operator screening a compound library can anticipate this problem while preparing the compounds dilution and identify possible interfering coloured compounds.

Also, a limitation of this method is the long incubation time (24 h) required to achieve a good signal window between positive and negative controls. As with the Alamar Blue assay [11], this could limit the determination of onset of compound activity. The long incubation time needed could be because schistosomula have low metabolism in comparison with cells undergoing constant multiplication by mitosis.

Praziquantel also has failed to produce a variable slope and the returned IC_50_ values from the analysis in both, microscopy and XTT assays, were classified as ambiguous, and the confidence intervals were very wide. However, this behaviour was previously reported in other studies [7, 11–14], as PZQ is highly active to adult worms, but it is poorly active in the larval stage. Despite that, parasites viability estimated by XTT assay after PZQ treatment was similar to microscopic evaluation (Fig. 5a). Some of the IC_50_ values presented here differ from previously published values for the same drugs. This could be due to parasite strain differences, time of compound exposure, or to the fact that the microscopy methodology we employed here was based on counting dead parasites stained by PI. Staining with PI is a straightforward and quantitative method to assess schistosomula viability that does not rely on subjective score attribution used elsewhere, which could lead to those IC_50_ differences [34].

Some efficient methods that do not rely on dyes to assess parasites viability are available and represent a complete and easy-to-use drug screening platform. Those computational methods do not suffer from compound coloration interference and allow viability assessment during different time-points. As for all methods, they all carry some limitations. The WormAssay is based on movement analysis of parasites, and it has been successfully employed only for adult worm screening [8]. Some methods that use automated image-based classification may require a systematic machine learning process [10], as well as very expensive devices, like the ImageXpressMicro HCS microscope [9]. Additionally, they might demand large computational force and data storage. Thus, the development of simple “just-add” dye-based assays that require minimal equipment are still comparatively utile, especially in lower-resource laboratories.

## Conclusions

In conclusion, we demonstrated that the XTT schistosomula viability assay developed by our group allows for the effective determination of parasite viability with high sensitivity. The protocol offers an easier and faster alternative to microscopic assessment, and with lower costs than fluorescence/luminescence-based assays. Moreover, we would like to highlight the importance of using guidelines, such as the NIH HTS Assay Validation manual [22], since it is unclear whether many of the proposed methodologies available for drug screening in *Schistosoma* reach the minimum criteria needed and are, sometimes, not reproducible in different laboratories.

## Abbreviations

CV: Coefficient of variation
DALYs: Disability-adjusted life years
DMSO: Dimethyl sulfoxide
FDA: Fluorescein diacetate
HTS: High-throughput screening
IVM: Ivermectin
MFQ: Mefloquine
NTS: Newly transformed schistosomula
PBS: Phosphate buffered saline
PI: Propidium iodide
PMS: Phenazine methosulfate
PZQ: Praziquantel
SD: Standard deviation
SW: Signal window
Z’: Z’ factor
